# Macrophages and the immune microenvironment in OPMDs: a systematic review of the literature

**DOI:** 10.3389/froh.2025.1605978

**Published:** 2025-05-13

**Authors:** Samuele Sutera, Olga Anna Furchì, Monica Pentenero

**Affiliations:** Department of Oncology, Oral Medicine and Oral Oncology Unit, University of Turin, Turin, Italy

**Keywords:** oral potentially malignant disorders (OPMD), cellular microenvironment, macrophages, carcinogenesis, oral leukoplakia (OL), oral epithelial dysplasia (OED), mouth diseases, precancerous conditions

## Abstract

**Background:**

In the presence of cancers, Tumor Associated Macrophages have a well-established role, but the literature provides limited evidence regarding their involvement in the onset and malignant transformation of Oral Potentially Malignant Disorders (OPMDs).

**Objectives:**

The present systematic review aimed to collect evidence on the presence and characterization of macrophages in the microenvironment of OPMDs.

**Data sources:**

PubMed, Scopus, EMBASE, Web of Science.

**Study eligibility criteria:**

Ex vivo or in silico human studies reporting original quantitative data on macrophage infiltration in OPMDs or Oral Epithelial Dysplasia (OED), published from 1990 onward.

**Results:**

Thirty-seven studies were included for qualitative analysis. Investigated OPMDs included: oral leukoplakia, oral lichen planus, oral lichenoid lesions, proliferative leukoplakia, oral submucous fibrosis, actinic cheilitis, chronic graft vs. host disease.

**Discussion:**

Even though the heterogeneity of data from the included studies prevents a meta-analysis, the reported results are quite consistent in supporting an increasing macrophage infiltration from normal mucosa to OPMDs, OED, and Oral Squamous Cell Carcinoma (OSCC). An M1 pro-inflammatory polarization is prevalent in OPMDs, with a shift toward an M2 pro-tumorigenic polarization in moderate-severe OED and OSCC. Several novel markers including STAT1, IDO, PD-L1, APOE, ITGB2 appear to be able to identify macrophage clusters involved in pro-inflammatory or pro-tumorigenic pathways.

**Conclusions:**

Evidence from the present review supports an active role of macrophages in regulating immune suppression, oncogenesis, and tumor progression in OPMDs and during the transition to OSCC. Future research should focus not merely on cell quantification and general M1/M2 polarization but rather on the expression of specific markers potentially linked to immunomodulatory pathways involved in oncogenesis.

## 1 Introduction

The potential role of microenvironment (ME) inflammation has been extensively investigated in carcinogenesis, tumor progression and potential implications for treatment across various malignancies, including Oral Squamous Cell Carcinoma (OSCC) (1, 2).

Moving to the onset and progression towards malignant transformation (MT) of Oral Potentially Malignant Disorders (OPMDs), the literature provides limited evidence and implications due to inflammatory infiltrate still needs to be fully elucidated (3). It would be valuable to understand whether the inflammatory ME has a role in promoting MT and whether its features and role are consistent among different OPMDs.

A band-like chronic inflammatory cell infiltrate in the superficial lamina propria (with a so-called lichenoid features) commonly accompanies premalignant and malignant oral lesions (4). Recently the potential role of the inflammatory infiltrate in promoting oral carcinogenesis has been highlighted and it has been hypothesized that OPMD-associated inflammation can represent a surrogate marker to diagnostically differentiate between Oral Epithelial Dysplasia (OED) and Oral Lichen Planus (OLP) and to predict MT (5).

Microenvironment is shaped by the dynamic interaction between inflammatory and non-inflammatory cells, along with a variety of mediators able to influence disease progression and the immune response (3, 6, 7). Evidences on the pivotal role of infiltrating macrophages (MΦ) in tumor development and progression lead to the establishment of the concept of Tumor Associated Macrophages (TAMs) (8–10). Moving to OPMDs, we are just laying the first foundations for a more in-depth knowledge of the potential role of the inflammatory infiltrate in OPMDs.

The present review aims to collect evidence from the literature about presence and characterization of MΦ infiltrate in OPMDs ME.

## 2 Methods

### 2.1 Data sources and search strategy

The present systematic review was conducted according to the Preferred Reporting Items for Systematic Reviews and Meta- Analyses (PRISMA) guidelines.

A thorough search strategy was designed together with an information specialist using a tailored query string for each database's specific requirements (see Supplementary Materials).

The following electronic databases were comprehensively searched (last search on January 2025): PubMed, a free database managed by the National Center for Biotechnology Information (NCBI) at the U.S. National Library of Medicine (NLM), Web of Science (WoS) a multidisciplinary citation database managed by Clarivate Analytics, Scopus and Embase databases managed by Elsevier.

We complemented the search by examining previous systematic reviews and literature reviews, as well as by bibliographic cross-referencing, to identify omitted studies.

### 2.2 Eligibility criteria

Inclusion criteria were:

• ex vivo human studies reporting original quantitative data on MΦ infiltration in OPMDs or OED
• in silico human studies
• Publications from 1990 onward

Exclusion criteria were:

• ex vivo human studies reporting descriptive information on MΦ infiltration in OPMDs or OED
• ex vivo non-human studies
• *in vitro* studies
• reviews, systematic reviews, conference abstracts, letters, or comments
• articles published in languages other than English

### 2.3 Study selection process

Studies retrieved from the databases were imported into a reference manager library (EndNote 21, Clarivate Analytics, Philadelphia, PA, USA). Duplicates were removed; the remaining records had a first-round screening performed by two independent raters. Disagreement was resolved by consensus. In the first round, records were screened by title and abstract. The level of agreement between the two raters will be assessed using Cohen's Kappa coefficient, which will be interpreted according to the scale proposed by Landis and Koch, which classifies agreement as slight, fair, moderate, substantial, or almost perfect (11). In the second round, the full-text papers were assessed. Articles fulfilling the eligibility criteria were selected for data extraction. Adjunctive papers retrieved during data extraction were included.

### 2.4 Quality assessment

The methodological quality of the included studies was assessed using the Study Quality Assessment Tools developed by the National Heart, Lung, and Blood Institute (NHLBI). These tools provide specific criteria for evaluating different study designs, including observational studies (12).

Each study was independently evaluated by two reviewers (MP and OAF), assigning 2 points for each criterion met (“yes”) and 0 points if not met (“no”). Responses marked as “unclear” or “partially met” were assigned 1 point, while “not applicable” criteria were excluded from the final score calculation.

A quality score (%) was calculated for each study using the following formula:

$$\mathrm{Quality}\;\;\mathrm{Score}\;(\text{\%})=\frac{N.\;\;\mathrm{of}\;\mathrm{yes}\;\mathrm{responses}+N.\;\mathrm{of}\;\mathrm{unclear}\;\mathrm{responses}}{\mathrm{Total}\;\mathrm{applicable}\;\mathrm{criteria}\times 2}\times 100$$

Based on the final score, studies were classified into three categories:

• High quality (≥80%)
• Moderate quality (60%–79%)
• Low quality (<60%)

Discrepancies between reviewers were resolved through discussion.

### 2.5 Data extraction

A standardized data extraction sheet was prepared and tested for clarity and consistency by three independent reviewers using a pilot set of three articles. Eligibility, validity, design information, OPMD included, sample size, technique used to identify MΦ, MΦ biomarkers assessed (i.e., CD) will be recorded on the extraction sheet for each study by two of the authors (MP and OAF). In case of discrepancies, we resolved any disagreements by discussion.

### 2.6 Data synthesis and analysis

A qualitative synthesis of the included studies was performed, summarizing key findings related to MΦ infiltration and focusing on their characterization and M1/M2 polarization. Quantitative data were extracted and, when possible, compared across studies. Given the heterogeneity of study designs, methods, and outcome measures, a meta-analysis was not performed. Instead, a descriptive approach was used to identify trends and patterns in MΦ infiltration and its potential association with different OPMDs and with disease progression towards MT.

## 3 Results

### 3.1 Literature search

**Figure 1:**
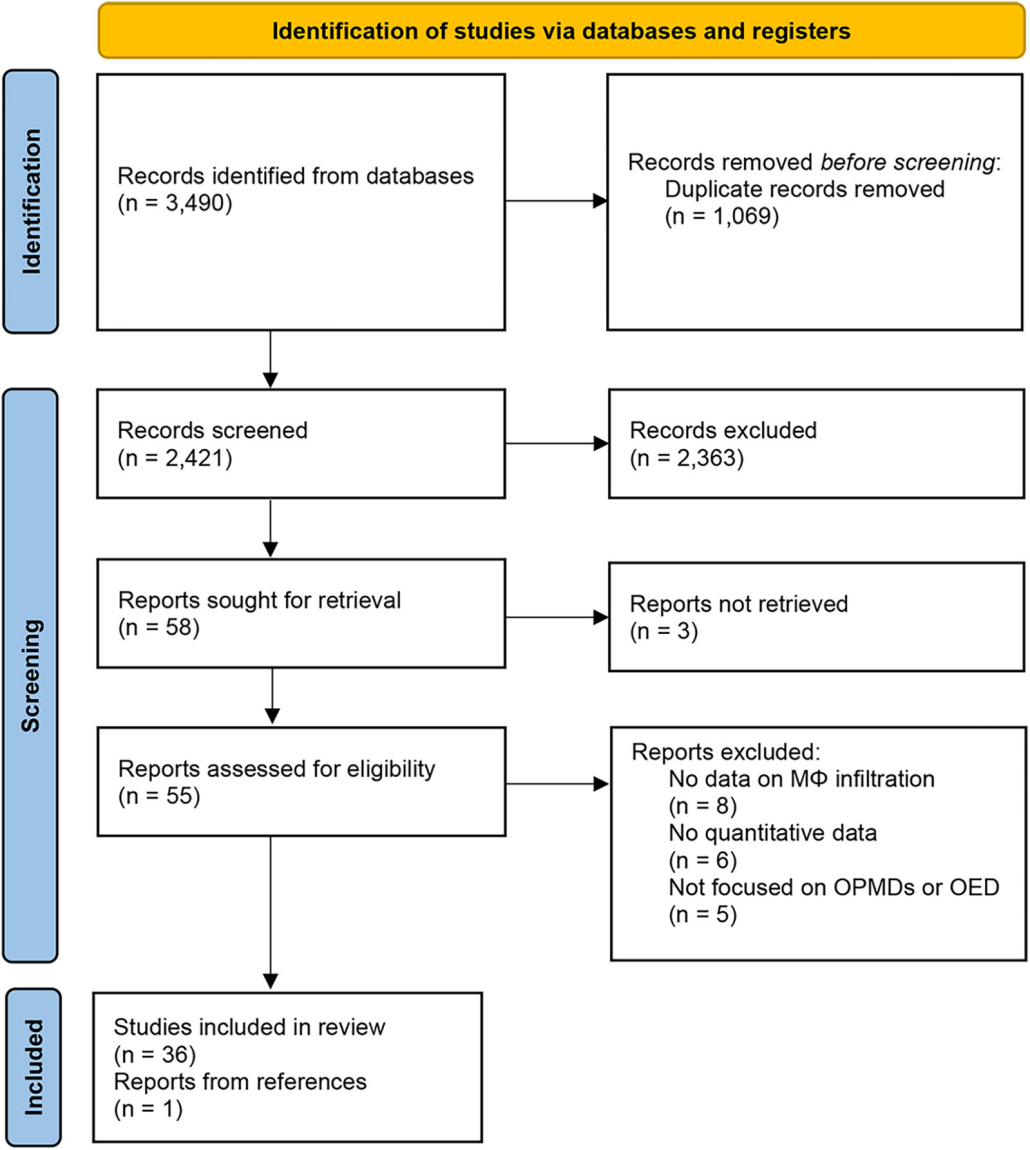
PRISMA flow diagram.

The selection process and reasons for exclusion are summarized in Figure 1. The search strategy in the databases resulted in 3,490 records. The last search was conducted on January 15th, 2025. Duplicates (1,069) and irrelevant studies (2,363) were excluded and a total of 58 studies were potentially eligible for inclusion. The agreement between the two reviewers during title and abstract screening was assessed using Cohen's Kappa coefficient, which yielded a value of 0.76 (95% CI: 0.52–0.99), indicating substantial agreement. The eligibility of 58 articles was assessed by full-text analysis, leading to the exclusion of 19 papers that did not meet the inclusion criteria and 3 papers not available, while one additional study retrieved from the references was included, resulting in a final review of 37 papers. Reasons for exclusion after full-text analysis are presented in Supplementary Table S1.

### 3.2 Description of the included studies

Almost all the retrieved studies investigating the presence of MΦ infiltration in tissue samples used IHC staining sometimes integrated with IF to co-localize multiple targets. All included studies reported quantitative results, although precise numerical values were not always provided, as some studies presented their findings solely through graphical data. The counting methods were highly heterogeneous across studies, leading to not comparable results that hindered the feasibility of a reliable meta-analysis. These methodological features of the included studies are presented in Supplementary Table S2.

A limited number of recent studies have conducted sc-RNAseq analyses (13–15) or explored transcriptional databases (RNA-seq) (16–18) from public repositories or original cohorts, assessing MΦ infiltration with CIBERSORTx (19).

#### 3.2.1 Oral epithelial dysplasia (OED)

Eleven studies assessed tissue samples harboring Oral Epithelial Dysplasia (OED), not always mentioning the associated OPMD (5, 17, 20–28).

An increasing trend in cell counts of CD68+ cells have been observed from OED (classified in a 3-grade scale and with a clinical diagnosis of OL) to OSCC. A significant increase was observed when comparing overall OED to OSCC, but pairwise assessments remained significant only when comparing mild OED to moderate and severe one. No significant increase was found between severe OED and OSCC (28). **IFN-γ+** MΦ were predominantly detected in OED rather than in OSCC and their presence was negatively correlated with the progression of oral dysplasia (28).

CD68+ MΦ infiltration is consistently increased in oral verrucous carcinoma (OVC), OED and OSCC compared to normal mucosa. No significant difference was observed between OED and OVC or OED and OSCC while the increasing CD68+ cells infiltration in OSCC compared to OED approaches significant values (*p* = 0.056) (27).

A couple of studies from the same Authors compared OSCC from the floor of the mouth, moderate to severe OED (all of them with a clinical diagnosis of OL from gingiva) and normal mucosa (24, 25). They found a significantly increased subepithelial infiltration of CD163+ MΦ in OSCC with regional/distant metastasis when compared to OED (24). Compared to normal mucosa, OED showed higher levels of CD163+ and iNOS+ cells, but only the increase in iNOS+ cells was statistically significant (24, 25). PD-L1+ infiltrating cells progressively and significantly increased from normal mucosa to OED and to OSCC, with positive correlation with the CD163+ cell count (24, 25).

The expression of MΦ markers CD68 and CD163 has been reported to be significantly and progressively increased from normal mucosa to OED (grading not reported) and OSCC (22).

More specifically another study found a progressive increase of both CD163+ and CD204+ cells from non-dysplastic OL to mild-moderate OED and from mild-moderate to severe OED, but no further increase was observed in OSCC compared to severe OED (23). Another study found a significant CD163+ increased infiltration only when comparing severe OED to normal mucosa (not the same for mild or moderate OED) but also in this cohort no further increase was observed in OSCC compared to severe OED (20).

The presence of CD163+ MΦ in the subepithelial compartment is significantly associated with moderate-severe OED, maintaining significance in multivariate analysis (26). Subepithelial PD-L1+ cell count was significantly associated to MT in a Cox proportional hazards model, but the exact nature of PD-L1+ subepithelial cells remains unclear, as only a subset co-expresses CD163 or CD8 (26).

In a cohort including Oral Lichen Planus (OLP) and Oral Lichenoid Lesions (OLL), CD163+ infiltration is highest in dysplastic OLL, followed by dysplastic OLP, non-dysplastic OLP and finally non-dysplastic OLL; paired analysis revealed a significant difference between these groups. Moreover, a positive correlation to β-catenin expression was observed (21).

In 10 cases of moderate to severe OED, intraepithelial CD163+/STAT1+ MΦ, mainly located in the subepithelial compartment, have also been observed within the epithelium and were correlated with similar infiltration patterns of CD8+ T cells (5). A gene ontology analysis from RNA-seq of the same cases showed immune signatures associated with immunosurveillance, lymphocyte infiltration, cytotoxic response, and surrogate markers of tumor-associated macrophages (5).

A CIBERSORTx analysis revealed that FibroEpithelial Polyps (FEP) (used as positive controls in this study) have higher CD68 expression compared to moderate-severe OED and early-stage OSCC, suggesting a decline in general macrophage presence. M1 macrophages appear significantly reduced in early-stage OSCC compared to FEP and moderate-severe OED. TAM infiltration is significantly increased in moderate-severe OED compared to FEP controls. The relative abundance of TAMs compared to antigen-presenting T cells (TAMsurr_TcClassII_ratio) also significantly increases in OED, indicating a shift towards an immunosuppressive microenvironment. No significant difference is observed between OED and early-stage OSCC (17).

#### 3.2.2 Oral leukoplakia (OL)

Oral Leukoplakia (OL) is the most commonly investigated OPMD, frequently compared to OSCC (13, 15, 16, 23, 28–35).

In OL the percentages of infiltrated CD68+ and CD80+ cells did not differ significantly by histopathological grade of OED (a 5 grade scale was used), but the number of CD163+ cells was significantly increased in mild-moderate and moderate OED compared to samples without OED (not the same for moderate-severe and severe OED) (35). Almost half (51.5%) of the CD163+ co-expressed STAT1 (35).

Progressive increases in CD68+ cell counts from OL to OSCC, with a correlation to OED severity, were also observed (28). IFN-γ+ MΦ were predominantly detected in OL rather than in OSCC, with a negative correlation to dysplasia progression (28).

When assessing the presence of CD68+ and CD163+ cells in normal mucosa, low-risk OL (non-dysplastic or with mild OED), high-risk OL (moderate-severe OED) and OSCC, the cell counts are all higher than in normal tissue and show a progressive increase. However, pairwise differences were not reported (34). Additionally, the expression of CD68 on MΦ positively correlated with the expression of SIRPα, and the expression of CD163 on MΦ negatively correlated with the expression of SIRPα (34). The infiltrating cells SIRPα + CD68+ and SIRPα+CD163+ in OL were higher than in normal mucosa and OSCC (34).

Subepithelial CD163+ and, to a lesser extent, CD206+ MΦ were present in OL, while CD204+ MΦ were absent. A high CD163+ cell infiltration was significantly associated with moderate-severe OED and CK13 loss, whereas CD206+ cells did not show the same correlation (32).

The same Authors compared biopsy samples from OL, where the presurgical diagnosis of intraepithelial lesion (i.e., OED) was confirmed in the surgical specimen, to biopsy samples in which the final pathological report revealed OSCC. The intraepithelial CD163+ infiltrate was significantly higher in the latter group, whereas no significant difference was observed in the subepithelial area (33).

Investigating a cohort of progressing and non-progressing OL, an overall significant increase of epithelial and sub-epithelial MΦ (CD68+) infiltration was observed in progressing OL comparted to both non-progressing OL and normal mucosa. Infiltrate in progressing OL is characterized by a significantly increased CD163/CD11c expression ratio only in the epithelial compartment. Moreover, progressing OL revealed a significantly increased epithelial/subepithelial CD163 expression ratio compared to non-progressing OL. ROC analysis identified increased CD68 and CD163 expression in the epithelial compartment and CD68 in the subepithelial compartment as potential predictors of MT within five years (31).

Gene expression and CIBERSORTx analyses of datasets examining OL, OL progressing to OSCC, and OSCC revealed significant differences in CD4+ T cell and MΦ infiltration across the various stages. Increased CD4+ T cell infiltration and a M0 to M2 MΦ polarization were observed in samples with a higher risk of progression from OL to OSCC, with gene expression analysis suggesting that elevated DHX9 and BCL2L12 expression in MΦ could be involved in MΦ polarization (16).

Comparing OL with moderate-severe OED to adjacent OSCC, by RNA-seq, an increasing proportion of immunoinhibitory Macro_NRG1 and Macro_APOE subclusters was noted throughout MT (15).

Zhang et al. identified six MΦ subsets, with Macro-IDO1 and Macro-PLA2G2D specifically enriched in OL concomitant with OSCC (OL-OSCC), suggesting a role in carcinogenesis. Notably, the proportion of Macro-PLA2G2D declined in OSCC compared to OL-OSCC, while Macro-IDO1 remained relatively abundant (13). Immunofluorescence staining for IDO1 and CD68 was employed to investigate the proportion of IDO1 + CD68+ MΦ both in the total cell population and among CD68+ cells. Results showed a significantly higher proportion in OL-OSCC compared to OL, while no significant difference was observed between OL-OSCC and OSCC in relation to total cells (13).

#### 3.2.3 Actinic cheilitis (AC)

In a cohort of AC, CD11c+ cells infiltrating the lamina propria were characterized by the intracellular expression of indoleamine 2,3-dioxygenase (IDO) and their cell count strongly correlated to the degree of epithelial atypia. Of interest MΦ (CD68+ cells) did not show IDO expression (36).

#### 3.2.4 Proliferative leukoplakia (PL)

A significantly higher density of FXIIIa+ and CD163+ MΦ was observed in the subepithelial area of PL compared to OL and control (30).

#### 3.2.5 Oral lichen planus (OLP), oral lichenoid lesions (OLL), graft vs. host disease (GvHD)

Results on OLP, OLL and GvHD are jointly reported as only 1 study specifically addressed OLL (37) and others included both OLP and OLL (21, 38) or OLP and GvHD (39, 40).

A high **CD68+** cells inflammatory infiltrate has been reported in OLP when compared to normal mucosa (41, 42). Even when compared to various grade of OED (in the presence of a clinical diagnosis of OL), OLP has a higher infiltration of CD68+ cells; lower when compared to OSCC (28).

Vered et al. found that CD163+ MΦ were expressed less than other pro-inflammatory biomarkers in OLP, while erosive variants of OLP showed increased expression of CD163 compared to hyperkeratotic forms (43).

Further analysis of CD68 and CD163 intra and sub epithelial expression in OLP and OLL also including positive controls (fibroepithelial polyps, FEP) revealed no significant differences in CD163+ infiltration across the three entities (38). However, CD68+ infiltration in subepithelial regions was significantly higher in OLP when compared to FEP or OLL and a positive correlation between CD68 and CD163 expression was observed in both OLP and OLL (38). No statistical significance was observed when associating CD68+ or CD163+ infiltrate with clinical variants of OLP and OLL (38).

IF/IHC assessments revealed that CD163+/STAT1+ MΦ were predominantly located in the subepithelial regions. In the intraepithelial compartment CD163+/STAT1+ cells accounted for 55.1% of all immunoreactive cells in OED and 34.3% in OLP (5).

Interestingly, an increased infiltration of CD68+ (40) and CD163+ (39) MΦ has also been reported in OLP and cGvHD when compared to normal mucosa, in the absence of differences between these two disorders (39, 40).

OLP tissue demonstrated a significantly higher density of both M1 (CD86+) and M2 (CD204+) MΦ, with a M1/M2 ratio of 1.67 (44). This is consistent with scRNA-seq data (GSE211630), which showed that MΦ numbers in OLP tissue were more than three times higher than in normal mucosa, although the proportion of MΦ within the inflammatory infiltrate was similar between OLP and normal tissues. Of note the top 5 upregulated genes were CXCL9, APOE, APOC1, CCL18 and CXCL10 (44).

CIBERSORTx analysis on datasets from OLP patients revealed a notable infiltration of M1 MΦ, activated dendritic cells, T follicular helper cells, and T regulatory cells, further supporting the presence of an inflammatory immune response in OLP (18).

ScRNA-seq analysis also showed that compared to normal mucosa, MΦ and conventional dendritic cells (cDCs) in OLP tissues exhibited enhanced overall inflammatory activity related to cell adhesion and antigen processing/presentation, thus contributing to infiltration of lymphocytes. Differential gene expression analysis and enrichment analysis showed in OLP blood a stronger cellular chemotaxis of macrophages. Finally, communications between T cell subtypes, myeloid cells and fibroblasts were increased and possibly following the ITGB2 pathway which dominated in OLP tissues (14).

RNA-seq data from 10 OLP patients revealed a characteristic inflammatory microenvironment with lymphocyte infiltration, T-cell regulation, and cytotoxic activity (5).

#### 3.2.6 Oral submucous fibrosis (OSF)

Five studies investigated OSF sometimes only including moderate to advanced stages of disease (45–49).

The less recent studies just investigated the presence of CD68+ finding out an increased MΦ infiltration (45, 47).

A study investigating only moderate to advanced OSF specimens compared to normal mucosa, found a significant increase in MΦ (CD68+ cells) densities only in the subepithelial connective tissue of OSF, while maintaining a proportional representation of immunocompetent cells (B-cells, T-cells and MΦ), without selective expansion of a particular immune cell type (45). The increased density of CD68+ cells was found in both the epithelium and the underlying lamina propria in a later study (47). The fact that these two studies, despite differing in design, one retrospective (45) and one prospective (47), report exactly the same mean values and standard deviations for macrophage counts in the control group raises concerns. This could indicate data reuse without disclosure, an identical sample of controls used in both studies, a transcription or publication error, or an extremely unlikely statistical coincidence. Given the improbability of obtaining identical results in an independent prospective study, further verification is warranted to assess potential methodological or reporting issues.

OSF and OL have been reported to have similar CD68+ MΦ infiltration while CD163+ cells were more represented in OL. No significant differences were found when comparing OL to OL concomitant with OSF, nor between OSF and OL concomitant with OSF (29).

Several studies have explored the role of M2-polarized MΦ in the onset and progression of OSF (48, 49). In OSF tissue higher amounts of CD68+ and ARG1+ cells (49) and abundant expression of CD163+, CD206+, CD209+ cells (48) were found if compared to normal mucosa. Bioinformatics analyses (GSE64216) confirmed ARG1significant overexpression in OSF (48, 49).

Arecoline, an active compound in areca nut, appears to contribute to M2 macrophage differentiation through various pathways, including the stimulation of fibroblasts to secrete IL-13 (48) and IL-4 (49).

Parekh et al. investigated the dynamic interplay between M1 and M2 MΦ across different stages of OSF and its malignant transformation. They observed a significant polarization towards M2 MΦ in advanced stages of OSF (Stages 3 and 4). Early-stage OSF (Stages 1 and 2) exhibited elevated M1 (CD11c+) MΦ expression, which shifted towards M2 (CD163+) in advanced stages, suggesting a transition to a pro-fibrotic, anti-inflammatory, and pro-tumorigenic environment. Furthermore, a significant upregulation of M2 (CD163+) MΦ compared to M1 (CD11c+) MΦ was also noted in the connective tissue of OSCC, along with a loss of epithelial M1 expression (46).

## 4 Discussion

In normal oral mucosa, MΦ are present in the subepithelial connective tissue but in lower numbers compared to other immunocompetent cells (T lymphocytes and dendritic cells) and they are only occasionally observed in the epithelium. Among inflammatory cells, MΦ have garnered considerable interest in recent years since they seem to play a critical role in tumor development and progression, so that MΦ associated to tumor progression are commonly referred as Tumor-Associated Macrophages (TAMs) (10). While typically sparse in mucosal and submucosal tissues, MΦ increase in both OPMDs and OSCC. These MΦ originate from resident macrophage and circulating monocytes, which are recruited by signals from epithelial cells and the surrounding stroma. **Dendritic cells (DCs) and MΦ** represent a first line of innate immune surveillance, recognizing pathogens or tissue damage; by presenting antigens to T cells they initiate and regulate adaptive immunity (50). Data from the literature investigating the expression of DCs in OPMDs are contrasting: some studies link OL, PL or high-grade OED to reduced CD1a+ Langerhans cell density (30, 51, 52), while others report an increase (53). Another study found no difference between OL samples with and without OED (54).

The immunocompetent cells, especially the MΦ and lymphocytes, are likely the main source of cytokine synthesis. Of interest, MΦ are characterized by an ambivalent role due to their potential polarization into two distinct phenotypic profiles: M1, generally associated with pro-inflammatory and anti-tumor activity, and M2, linked to anti-inflammatory and pro-tumor functions.

Immunosuppressive tumor microenvironment (iTME) plays a key role in carcinogenesis, and some MΦ subsets are associated with iTME generation. However, the sub-population characterization of MΦ in oral carcinogenesis remains largely unclear (13).

TAMs display remarkable plasticity within the TME and may transform from one phenotype to another and they always present a mixture of M1-like and M2-like phenotypes (55).

Classically activated (**M1)** MΦ are induced in response to IFN-γ and lipopolysaccharides and they are characterized by the production of high levels of pro-inflammatory cytokines including tumor necrosis factor-α (TNF-α), interleukin IL-1, IL-6, IL-12, IL-23 and inducible nitric oxide synthase (iNOS), which play a crucial role in their anti-tumor activity. Notably, iNOS plays a dual role: in early stages, it promotes inflammation and anti-tumor immunity, while in advanced tumors, it contributes to immune suppression and tumor progression. Alternatively activated (**M2)** MΦ mainly secrete molecules involved in processes such as angiogenesis, tissue remodeling, and tumor progression: IL-10, arginase (ARG), and transforming growth factor β (TGF-β) to suppress the inflammatory response and upregulating mannose receptors, scavenger receptors, and angiogenic factors like vascular endothelial growth factor (VEGF), while exhibiting low levels of pro-inflammatory cytokines (8). M1 TAMs can recruit CD8+ cytotoxic T cells, while M2 TAMs predominantly attract CD4+ T cells, particularly Tregs (56).

Different **markers** are associated with M1 or M2 polarization (57, 58) as shown in Table 1. It is known that the M2-like phenotype predominates in TAMs with an established tumor-promoting effect and M1-like phenotype are conventionally known for their anti-tumor functions, including the induction of inflammation and direct tumor cell attack. However, recent studies suggest that, under specific conditions, they may also contribute to tumor progression (59). Furthermore, TAMs expressing different markers have been observed to localize in different regions within the TME, and it could be related to their specific functions. Namely, CD163+ cells are distributed throughout the stroma, whereas CD204+ and CD206+ cells are predominantly concentrated near the tumor nest (60).

**Table 1:** M1/M2 polarization markers.

Macrophage Polarization	Marker	Function/Description
M1 (Pro-inflammatory, Anti-tumor)	CD68	General macrophage marker (not specific for M1 or M2)
	CD11c	Integrin primarily expressed on dendritic cells, involved in cell adhesion, antigen presentation, and immune activation
	CD80 (B7-1)	Co-stimulatory molecule associated with classical M1 polarization
	CD86 (B7-2)	Co-stimulatory molecule upregulated in M1 polarization
	HLA-DR (MHC class II)	Highly expressed in M1 polarization
	iNOS (inducible nitric oxide synthase, NOS2)	Key enzyme in NO production and M1 function
	TNF-α	Pro-inflammatory cytokine secreted by M1 MΦ
	IL-12	Cytokine that promotes Th1 responses, typically produced by M1 MΦ
M2 (Anti-inflammatory, Tissue-remodeling, Pro-tumor)	CD163	Scavenger receptor, commonly used as an M2 marker
	CD204	Scavenger receptor, mainly expressed by M2 MΦ
	CD206 (Mannose Receptor, MRC1)	Classic M2 marker involved in endocytosis and tissue remodeling
	Arginase-1 (ARG1)	Enzyme associated with M2 function, suppresses inflammation
	IL-10	Anti-inflammatory cytokine secreted by M2 MΦ
	TGF-β	Immunosuppressive cytokine, involved in fibrosis and tumor progression
	CCL18	Chemokine produced by M2 MΦ, found in tumor microenvironment

The recruitment of MΦ could be an early event in the carcinogenesis process, which could lead to the initial dense infiltration of both M1 and M2 MΦ. Therefore, an overall assessment could not be able to detect differences due to macrophage polarization rather than to the overall cell count. When comparing macrophage infiltration between different steps in the carcinogenesis process (e.g., OED and OSCC), an overall assessment based of a pan-macrophage marker (CD68) could not be able to differentiate such conditions (27). Additionally, since polarization exists on a spectrum rather than a strict dichotomy, using multiple markers increases accuracy. To accurately quantify the proportion of M1 and M2 MΦ, a combination of markers should be used (e.g., CD80/CD86 for M1, CD163/CD206 for M2).

Recent advances in techniques including scRNA-seq and spatial transcriptomics have made it possible to simultaneously obtain spatial organization information and transcriptome data, providing a comprehensive spatiotemporal perspective on gene expression within a specific tissue or throughout disease progression (61). A couple of studies applied such techniques on sampling from the same patient and simultaneously containing a normal region, OL harboring moderate-severe dysplasia (OED-OSCC) and OSCC (13, 15). This method is of interest as pairwise sampling allows to reduce heterogeneity when comparing sampling (62).

The aim of the present review is to improve knowledge of the role of MΦ in OPMDs; this implied the inclusion of heterogeneous studies where different OPMDs and different MΦ markers have been investigated. In addition to this, clear numerical data were not always available, making a meta-analysis approach unfeasible. Even with such limitations, a comprehensive analysis of these studies, including 1,573 samples from OPMDs or OSCC, reveals quite consistent results, describing differences (often significant) in MΦ infiltration between normal mucosa, OPMDs, OED, and OSCC. The present review, excluding *in vitro* studies, could have missed research investigating the mechanisms through which MΦ influence the immune microenvironment and contribute to carcinogenesis.

When considering tumor immunology, OPMDs could represent the equilibrium phase as defined by the concept of cancer immunoediting (26), but key inflammatory mediator(s) able to modulate the progression of OPMDs to OSCC have not yet been identified.

In the presence of OPMDs, MΦ may predominantly exhibit an anti-tumorigenic phenotype, aimed at counteracting MT, this could be consistent with an increasing M2 prevalence positively correlated with the grade of OED, while in earlier stages M1 have been reported to be present (35); CD163+ macrophages in oral leukoplakia co-express active STAT1 and suggest that the CD163+ macrophages possess an M1 phenotype in a Th1-dominated microenvironment. Consistently, RNA-seq and **Gene Ontology** analyses revealed in moderate-severe OED an inflammatory microenvironment primarily characterized by altered expression of genes related to immunosurveillance, lymphocyte infiltration, cytotoxic response, and surrogate markers of tumor-associated MΦ (5). This could align with a clinical scenario where OL does not progress to MT, at least in the short term, possibly due to MΦ polarized toward an anti-tumorigenic phenotype. If, as suggested by Mori, CD163+ and STAT1+ characterize MΦ infiltrating OL before transformation, this may indicate that MΦ are actively working to prevent malignant progression. The observed presence of infiltrated Th1 cells (CD4+ T cells CXCR3+ and CCR5+), producing IFN and affecting the phenotype of CD163+ MΦ in OL could be consistent with a ME trying to contrast the progression of OL. However, since Mori selected OL cases without considering their eventual transformation, the long-term clinical trajectory remains uncertain. Finally, the increased presence of CD163+ MΦ and intraepithelial CD4+ Th1 cells observed in the presence of moderate OED, compared to samples without dysplasia, could be associated with an increased immunogenicity of dysplastic keratinocytes (35).

Transforming OL showed an increased or decreased CD163+ MΦinfiltration in the epithelial or subepithelial compartment respectively, while no differences for CD11c+ MΦ infiltration were observed related to MT in OL (31). In transforming OL an increased infiltration of CD163+ MΦ and an M2 polarization (inferred based on the CD163/CD11c expression ratio) were observed only in the epithelial compartment (31). It is important to highlight that the study included OL cases with MT occurring within a five-year timeframe. This extended period of transformation does not allow to exclude the possibility of a gradual shift from M1 to M2 polarization, where MΦ may initially display an M1 phenotype to prevent malignant transformation. The presence and grade of OED was associated with macrophage infiltration (31). The presence of CD163+ MΦ in the epithelial compartment has also been reported to precede MT by up to two years (63) and it has been proposed as a red flag in cases where incisional biopsy results appear negative for OSCC, but there is a high clinical suspicion (33). In studies nonspecifically addressing MΦ, the presence of an inflammatory microenvironment has been observed in 57% of non- dysplastic OL that later underwent MT (64). Similarly, the inflammatory microenvironment associated to PL is not a recent discovery. Silverman, shortly after identifying Proliferative Verrucous Leukoplakia, reported an “inflammatory infiltrate in the connective tissue quite variable, ranging from mild and diffuse to dense subepithelial clustering” (65). This finding has been confirmed in subsequent case studies and is more common in the early stages, where multiple white plaque-like lesions may present with chronic sub-epithelial inflammation in the absence of OED (66). Such evidence suggests a potential role for inflammatory microenvironment not limited to OPMDs with a recognized inflammatory pathogenesis (e.g., OLP).

Notably, although most studies report an increase in CD163+ cell infiltration in the transition from high-grade OED to OSCC, a couple of studies do not confirm this finding (23, 49). Kouketsu et al. did not find a significant difference when analyzing CD204+ cells either; conversely, a significant increase was observed between non-dysplastic OL and severe OED (including carcinoma *in situ*). It could be speculated whether merging severe OED and carcinoma *in situ* may have affected such results (23). Nevertheless, the absence of significant differences between high-grade OED and OSCC could reflect early M2 polarization occurring in high-grade OED, thus favoring MT rather than acting as an immune effector against malignant progression. Looking at the IHC data from Yuan and Li, the progressive increase in CD163+ cell expression from moderate to severe OED to OSCC results in a statistically significant difference only when comparing severe OED or OSCC to normal mucosa, but not when addressing the transition from OED to OSCC (49).

In OPMDs, chronic inflammation may recruit CD11c+ monocytes that differentiate into macrophages (CD68+) under the influence of cytokines such as M-CSF, GM-CSF, and TGF-β. Subsequent activation of NF-κB in these macrophages can further regulate their inflammatory and immunosuppressive functions. Moreover, NF-κB induces the expression of indoleamine 2,3-dioxygenase (IDO) expression in MΦ (67). Zhang et al. identified Macro-IDO1 and Macro-PLA2G2D as dominant subsets in OL-OSCC and almost not existent in distant normal mucosa, thus conceivably involved in carcinogenesis (13). IDO is an intracellular enzyme that is primarily expressed in antigen presenting cells such as in dendritic cells (CD11c+) and MΦ (CD68+) and represents a mechanism of acquired immune tolerance in cancer (68). The expression of **IDO** in OPMDs has been investigated in a couple of studies with the same laboratory methods (double/multiple IF). In AC, CD68+ cells did not show IDO expression (36). Similarly, in OL, IDO1+ macrophages were nearly absent, but their infiltration significantly increased in OL associated with OSCC (13). Moreover the same study reported a positive relationship between the proportions of IDO1 + CD68+ cells (IDO1 + macrophages) and the PD-1 + CD3 + CD8+ cells (exhausted CD8+ T cells) in OL associated to OSCC and OSCC (13).

Further assessments in mice revealed that IDO1 inhibitors significantly reduces 4NQO induced oral carcinogenesis. These findings suggest that Macro-IDO1 is a key macrophage sub-cluster potentially associated to the shift of OL to OSCC (13).

In OSCC IDO1 + macrophages were strongly positively correlated with IL6/JAK/STAT3 signaling (69). IL-6/JAK/STAT3 and NF-κB signaling form a feed-forward loop, promoting sustained inflammation and disease progression. IL-6-induced STAT3 activation enhances NF-κB signaling by increasing pro-inflammatory cytokines such as IL-6 itself, TNF-α, and IL-1β while also suppressing NF-κB inhibitors such as SOCS proteins.

**IFN-γ+** MΦ were predominantly detected in OL rather than in OSCC and their presence was negatively correlated with the progression of oral dysplasia in OL (28). STAT is an interferon-inducible product, and the activation of **STAT1** in tumor-associated MΦ (TAMs) can lead to the upregulation of both inducible nitric oxide synthase (iNOS) and Arginase I. These enzymes play significant roles in suppressing T cell function—iNOS by generating nitric oxide, which can directly inhibit T cell activity (70), and Arginase I by depleting L-arginine, an amino acid essential for T cell proliferation and function (71).

Notably, a study comparing OED and normal mucosa reported an increased presence of both CD163+ and iNOS+ cells, though only the rise in iNOS+ cells was statistically significant (24). This finding may indicate a predominant M1 polarization in the early stages of carcinogenesis.

**CD163** **+** **STAT1+** MΦ likely represent a plastic or intermediate phenotype, balancing between inflammatory and immunosuppressive functions. They likely represent a potential target for studies investigating OPMDs and particularly conditions characterized by chronic inflammation.

In moderate/severe OED CD163 + STAT1+ were predominantly located beneath the epithelium, but they have also been observed as part of the intraepithelial compartment. In both these locations they seem to account for almost half of immunoreactive cells: 51.5% and 55.1% in the subepithelial and epithelia compartment respectively (5, 35). Both studies found a positive correlation between the presence of CD163+/STAT1+ cells and the distribution of CD4+ cells, supporting the notion that Th1 CD4+ T cell-derived IFN-γ may contribute to their recruitment and activation.

The pro-tumorigenic activity of CD163+ MΦ was shown to be dependent on the presence of nuclear **NF-κB** (72). Few studies jointly assessed CD163+ cells and the expression of nuclear NF-κB; among them Vered et al. reported an almost complete lack of nuclear expression of NF-κB within the inflammatory cells, indicating a proinflammatory rather than a protumorigenic direction of the CD163+ MΦ in OLP (43).

The **PD-1/PD-L1** expression is positively correlated with macrophage infiltration and is often associated with higher concentrations of CD4+ and CD8+ T cells in the immune infiltrate. The PD-1/PD-L1 associated pathway is a key immune checkpoint mechanism that regulates immune tolerance and immune evasion in diseases like cancer (73). MΦ, particularly those polarized toward the M2 phenotype, can actively contribute (up to 14%–32% of PD-L1 expression) to immune suppression through PD-L1 upregulation leading to T cell exhaustion and immune evasion. Several studies consistently suggest that OPMDs may evade the host immune system by PD-L1 expression on not only dysplastic epithelial cells but also the recruited subepithelial microenvironment.

A joint investigation of the expression of CD163 and PD-L1 revealed that more than 90% of CD163+ cells were distributed in the superficial lamina propria (similarly to CD8+ cells). In a mean follow-up of 45.6 months, subepithelial PD-L1+ cell count was found to be a significant risk factor for MT. Though, the characteristics of subepithelial PD-L1+ cells remain to be elucidated as double IF revealed the origin of a limited number of subepithelial cells co-expressing PD-L1, being MΦ (CD163+) 16.6% or CD8+ cells 14.1% (26). Of interest a phase II nonrandomized controlled trial assessing the safety and efficacy of nivolumab in PL patients showed a 2-y cancer-free survival of 73%, consistent with only potential anti-PD-1 activity (74).

Sun et al. observed increasing immunosuppressive macrophage subclusters, specifically Macro_NRG1 and Macro_APOE, when comparing OED-OSCC to OSCC. This suggests that these cells may play a role in MT (15).

The detection of APOE+ MΦ (Macro_APOE) in OPMDs suggests potential involvement in immune suppression and disease progression. These cells were frequently found alongside other immunosuppressive populations as NRG1+ MΦ or regulatory T cells (Tregs) expressing NFRSF4, reinforcing their potential role in immune evasion (15). In other cancers, APOE+ TAMs seem to resemble lipid-associated MΦ (LAMs) and exhibit an M2-like immune profile Braun et al. (75)).

Single-cell analysis also suggests that tissue-resident-like MΦ (Macro_SELENOP) may give rise to Macro_APOE/NRG1, particularly during the transition from OED-OSCC to OSCC (15). This is in keeping with findings from other malignancies, such as renal cell carcinoma, where APOE-enriched M2-like TAMs have been associated with disease progression (75). Finally, high numbers of MΦ co-expressing APOC1 and APOE were found in metastatic lymph nodes of esophageal squamous cell carcinoma, a cancer with a metastatic pattern similar to OSCC (76). The fact that APOE+ macrophages are consistently linked to tumor progression across multiple cancers suggests they may have a conserved, pro-tumorigenic function.

In OLP activated cytotoxic (CD8+) T-lymphocytes are thought to interact with other inflammatory cells such as helper (CD4+) subpopulations, Langerhans cells (CD1a+), and MΦ (CD68+), as well as basal keratinocytes, leading ultimately to keratinocyte apoptosis (77). The renowned pathogenetic role of chronic inflammation in OLP makes it an intriguing disease to investigate and compare to other OPMDs. In two studies both OLP and OLL were included (21, 38). In the absence of OED, both studies reported a higher CD163+ infiltrate in OLP compared to OLL, but the difference was significant in only one of them. Since STAT is an interferon-inducible product, it would have been interesting to examine the potential co-expression of CD163 and STAT1. When compared to OL a significant increase in CD68+ and IFN-γ+ cells was observed as expected, but no other characterization was performed in the study (28). It has been hypothesized that long-term constant use of steroids may contribute to the possible MT in OLP (77), this appears to be consistent with the described upregulation of CD163+ MΦ by glucocorticoids (78). Considering the potential pro-tumorigenic interaction between NF-κB and CD163+ cells, this hypothesis could suggest that alternative therapeutic agents could modulate the immune response in OLP attenuating the inductive effect of the epithelial NF-*κ*B on the interface inflammatory response and regulating the action of TGF-β (43).

In OLP submucosa, MΦ but not T-lymphocytes were identified by merged fluorescent double staining as TRPA1 immunopositive (79), this led to hypothesize that macrophage-derived TRPA1 may contribute to the transition from early immune responses to a chronic inflammatory condition (80). Li et al. explored cell-cell interactions in OLP finding the ITGB2 pathway enriched in T cells, myeloid cells, and fibroblasts when compared to normal mucosa (14). This pathway influences immune cell adhesion, migration, and activation and is supposed to positively regulates cellular adhesion of tissue MΦ. ITGB2 encodes for integrin LFA-1, which plays a pivotal role in T cell and possibly MΦ chemotaxis and tissue infiltration. The importance of the ITGB2 pathway in MΦ function is emphasized by a positive correlation between ITGB2 expression and CD163+ MΦ infiltration in esophageal carcinoma where scRNA-seq analysis indicated a progressive increase in ITGB2 with the acquisition of a tumor-promoting phenotype (81). Similarly, in ovarian cancer, ITGB2 is upregulated when compared to normal tissue and a high ITGB2 expression correlate positively with the infiltration of immune cells, particularly of M2 macrophages (82).

Current research on MΦ in the OPMD microenvironment is still scanty and presents potential gaps. Only one study retrospectively compared progressing and non-progressing OLs, trying to identify microenvironment features associated with MT (31). The lack of comparative studies assessing different OPMDs while properly accounting for the presence and grade of dysplasia, which should be considered a major confounding factor, represents a significant gap in the current literature. Such an approach might offer valuable insights, as the nature and composition of the inflammatory infiltrate could identify common inflammatory signatures related to carcinogenesis or, conversely, could differentiate OPMDs sharing similar clinical features. Moreover, the anatomical subsite could represent a potential bias related to risk habits (e.g., the mucobuccal fold in the case of tobacco or betel quid chewing, and the gingiva in the presence of plaque-related inflammation).

Looking at methods and data reporting, heterogeneity in study design and scoring methods limits reproducibility and the development of MΦ-based prognostic models, and the lack of quantitative data reporting prevents the possibility of performing meta-analyses. Finally, the assumption of anti-inflammatory or immunomodulating drugs was rarely considered as an exclusion criterion or at least as a confounding factor.

## 5 Conclusions

Despite the observed heterogeneity among the included studies, evidence from the present review supports an active role of MΦ in regulating immune suppression, oncogenesis, and tumor progression in OPMDs and during the transition to OSCC. They appear therefore to be the direct precursors of TAM subsets observed in other malignancies. Future research should focus not merely on cell quantification and general M1/M2 polarization but rather on the expression of specific markers potentially linked to immunomodulatory pathways involved in oncogenesis, particularly in light of the capabilities offered by new technologies such as scRNA-seq and CIBERSORTx.

## Data Availability

The original contributions presented in the study are included in the article/Supplementary Material, further inquiries can be directed to the corresponding author.
