# Supplementary material for: Macrophages and the immune microenvironment in OPMDs: a systematic review of the literature
Source: Front Oral Health. 2025 May 13;6:1605978. doi: 10.3389/froh.2025.1605978 (PMC12106459; doi:10.3389/froh.2025.1605978)
Supplement: Supplementary file 1 [file Datasheet1.docx]

# Query strings used for literature search

**Pubmed**

("Macrophages"[Mesh] OR macrophag*[tiab] OR tumor-associated-macrophag*[tiab]) AND (("Mouth Neoplasms"[Mesh] OR mouth-tumor*[tiab] OR mouth-tumour*[tiab] OR mouth-neoplas*[tiab] OR mouth-cancer*[tiab] OR mouth-carcinoma*[tiab] OR oral-tumor*[tiab] OR oral-tumour*[tiab] OR oral-neoplas*[tiab] OR oral-cancer*[tiab] OR oral-carcinoma*[tiab] OR oral-squamous-cell-carcinoma*[tiab] OR oral-potentially-malignant-disease*[tiab] OR oral-potentially-malignant-disorder*[tiab] OR OPMD[tiab] OR "Lichen Planus, Oral"[Mesh] OR oral-lichen*[tiab] OR "Leukoplakia, Oral"[Mesh] OR oral-leukoplaki*[tiab] OR mouth-leukoplaki*[tiab] OR oral-leucoplaki*[tiab] OR mouth-leucoplaki*[tiab] OR proliferative-verrucous-leukoplaki*[tiab] OR oral-dysplasi*[tiab] OR oral-erythroplaki*[tiab] OR mouth-erythroplaki*[tiab] OR "Actinic cheilitis" [Supplementary Concept] OR actinic-cheilitis[tiab] OR "Oral Submucous Fibrosis"[Mesh] OR oral-submucous-fibros*[tiab] OR mouth-submucous-fibros*[tiab]) OR (("Keratosis, Actinic"[Mesh] OR actinic-keratos*[tiab] OR "Lupus Erythematosus, Systemic"[Mesh] OR systemic-lupus-erythemat*[tiab] OR systemic-lupus[tiab] OR "Lupus Erythematosus, Discoid"[Mesh] OR discoid-lupus-erythemato*[tiab] OR discoid-lupus[tiab] OR "Graft vs Host Reaction"[Mesh] OR graft-versus-host[tiab] OR allogeneic-disease*[tiab] OR GVH disease*[tiab]) AND ("Mouth Diseases"[Mesh] OR mouth-disease*[tiab] OR mouth-patholog*[tiab] OR oral-disease*[tiab] OR oral-patholog*[tiab] OR oral-potentially-malignant-disease*[tiab] OR oral-potentially-malignant-disorder*[tiab] OR oral-premalignan*[tiab] OR oral-pre-malignan*[tiab] OR oral-precancer*[tiab] OR oral-pre-cancer*[tiab] OR oral-precarcinoma*[tiab] OR oral-pre-carcinoma*[tiab])))

**Scopus - Embase**

('macrophage'/exp OR macrophag*:ti,ab,kw OR tumor-associated-macrophag*:ti,ab,kw) AND (('mouth tumor'/exp OR mouth-tumor*:ti,ab,kw OR mouth-tumour*:ti,ab,kw OR mouth-neoplas*:ti,ab,kw OR mouth-cancer*:ti,ab,kw OR mouth-carcinoma*:ti,ab,kw OR oral-tumor*:ti,ab,kw OR oral-tumour*:ti,ab,kw OR oral-neoplas*:ti,ab,kw OR oral-cancer*:ti,ab,kw OR oral-carcinoma*:ti,ab,kw OR oral-squamous-cell-carcinoma*:ti,ab,kw OR 'oral potentially malignant disorder'/exp OR oral-potentially-malignant-disease*:ti,ab,kw OR oral-potentially-malignant-disorder*:ti,ab,kw OR OPMD:ti,ab,kw OR 'oral lichen planus'/exp OR oral-lichen*:ti,ab,kw OR 'oral leukoplakia'/exp OR oral-leukoplaki*:ti,ab,kw OR mouth-leukoplaki*:ti,ab,kw OR oral-leucoplaki*:ti,ab,kw OR mouth-leucoplaki*:ti,ab,kw OR proliferative-verrucous-leukoplaki*:ti,ab,kw OR oral-dysplasi*:ti,ab,kw OR 'oral erythroplakia'/exp OR oral-erythroplaki*:ti,ab,kw OR mouth-erythroplaki*:ti,ab,kw OR 'actinic cheilitis'/exp OR actinic-cheilitis:ti,ab,kw OR 'oral submucous fibrosis'/exp OR oral-submucous-fibros*:ti,ab,kw OR mouth-submucous-fibros*:ti,ab,kw) OR (('actinic keratosis'/exp OR actinic-keratos*:ti,ab,kw OR 'systemic lupus erythematosus'/exp OR systemic-lupus-erythemat*:ti,ab,kw OR systemic-lupus:ti,ab,kw OR 'discoid lupus erythematosus'/exp OR discoid-lupus-erythemato*:ti,ab,kw OR discoid-lupus:ti,ab,kw OR 'graft versus host reaction'/exp OR graft-versus-host:ti,ab,kw OR allogeneic-disease*:ti,ab,kw OR GVH disease*:ti,ab,kw) AND ('mouth disease'/exp OR mouth-disease*:ti,ab,kw OR mouth-patholog*:ti,ab,kw OR oral-disease*:ti,ab,kw OR oral-patholog*:ti,ab,kw OR oral-potentially-malignant-disease*:ti,ab,kw OR oral-potentially-malignant-disorder*:ti,ab,kw OR oral-premalignan*:ti,ab,kw OR oral-pre-malignan*:ti,ab,kw OR oral-precancer*:ti,ab,kw OR oral-pre-cancer*:ti,ab,kw OR oral-precarcinoma*:ti,ab,kw OR oral-pre-carcinoma*:ti,ab,kw)))

**Web of Science**

TS=((macrophag* OR tumor-associated-macrophag*) AND ((mouth-tumor* OR mouth-tumour* OR mouth-neoplas* OR mouth-cancer* OR mouth-carcinoma* OR oral-tumor* OR oral-tumour* OR oral-neoplas* OR oral-cancer* OR oral-carcinoma* OR oral-squamous-cell-carcinoma* OR oral-potentially-malignant-disease* OR oral-potentially-malignant-disorder* OR OPMD OR oral-lichen* OR oral-leukoplaki* OR mouth-leukoplaki* OR oral-leucoplaki* OR mouth-leucoplaki* OR proliferative-verrucous-leukoplaki* OR oral-dysplasi* OR oral-erythroplaki* OR mouth-erythroplaki* OR actinic-cheilitis OR oral-submucous-fibros* OR mouth-submucous-fibros*) OR ((actinic-keratos* OR systemic-lupus-erythemat* OR systemic-lupus OR discoid-lupus-erythemato* OR discoid-lupus OR graft-versus-host OR allogeneic-disease* OR GVH disease*) AND (mouth-disease* OR mouth-patholog* OR oral-disease* OR oral-patholog* OR oral-potentially-malignant-disease* OR oral-potentially-malignant-disorder* OR oral-premalignan* OR oral-pre-malignan* OR oral-precancer* OR oral-pre-cancer* OR oral-precarcinoma* OR oral-pre-carcinoma*))))
